# Investigation of Volatile Compounds in Varied Types of Gardenia White Teas Utilizing HS–SPME–GC–MS and Multivariate Analysis

**DOI:** 10.3390/metabo15120785

**Published:** 2025-12-05

**Authors:** Shenghong Zheng, Chunju Peng, Qi Huang, Ke Zhang, Zhengwen Niu, Guanghui Zeng, Huajing Kang, Hongling Chai

**Affiliations:** Wenzhou Key Laboratory of Early-Sprouting Tea Breeding, Wenzhou Academy of Agricultural Sciences, Wenzhou 325006, China; zsh1418@126.com (S.Z.); chunjupeng@wzvcst.edu.cn (C.P.); huangqi@wzvcst.edu.cn (Q.H.); zhangke@wzvcst.edu.cn (K.Z.); niuzhengwen@wzvcst.edu.cn (Z.N.); zengguanghui@wzvcst.edu.cn (G.Z.)

**Keywords:** gardenia white tea, HS-SPME-GC-MS, volatile compounds, multivariate analysis

## Abstract

Gardenia tea is esteemed for its delicate and fragrant aroma. **Background**: However, there is a scarcity of studies focused on the aromatic properties of gardenia-scented white tea, particularly regarding how these aroma profiles evolve over different storage durations. **Methods**: This research sought to analyze the volatile compounds present in gardenia white tea through headspace solid-phase microextraction gas chromatography–mass spectrometry (HS-SPME-GC-MS) alongside multivariate analysis techniques. **Results**: Findings indicated that the main chemical categories found in newly white tea (NWT), aged white tea (AWT), gardenia newly white tea (GNWT), and gardenia aged white tea (GAWT) included esters, terpenoids and ketones. The multivariate analysis pinpointed 11 significant volatile compounds (such as linalool, [(*Z*)-non-6-enyl] acetate, and (*E*)-non-4-enal) and an 10 additional key volatile compounds (including linalool, [(*Z*)-non-6-enyl] acetate, and 1-isothiocyanato-3-(methylthio)-2-Propane) that had variable importance in projection (VIP) values exceeding 2 and odor activity values (OAVs) greater than 1. These compounds effectively distinguished the aroma profiles of GNWT from NWT and GAWT from AWT. Notably, the levels of these compounds were significantly elevated in GNWT and GAWT compared to their NWT and AWT counterparts. Additionally, three volatile compounds in GNWT and six in GAWT showed a decline in concentration relative to NWT and AWT. **Conclusions**: These compositional differences are suggested to clarify the aromatic distinctions between gardenia-scented white tea and its white tea base. The outcomes of this study will contribute to a deeper chemical understanding of the unique aroma of gardenia white tea, providing a theoretical basis for assessing quality and developing products based on different storage periods.

## 1. Introduction

Scented tea, a prominent category of reprocessed tea in China, is renowned for its exquisite fragrance and harmonious flavor, attracting a devoted following [1,2]. The production of scented tea primarily involves mixing tea leaves with fresh blossoms, allowing for the infusion of aromatic compounds [3]. Jasmine green tea is currently the most well-known and extensively studied type [4,5]. Other significant varieties include rose black tea and osmanthus oolong tea [6], though research on additional scented teas remains limited. *Gardenia jasminoides* Ellis, a key fragrant plant in China, is commonly found in regions like Sichuan, Jiangsu, and Zhejiang province [7]. It is recognized for its rich array of phytochemicals and has been valued in traditional medicine for many years. Recent studies have highlighted its potent antioxidant [8], anti-inflammatory [9], and liver-protective properties [10], largely due to iridoid glycosides like geniposide and carotenoids such as crocins [11,12]. Presently, gardenias are also incorporated into tea production. Prior research has explored the processing methods, varieties, grades, and aromatic elements of Gardenia teas [13,14,15,16,17]. However, there is a lack of comprehensive studies on the volatile aromatic compounds in gardenia white tea. Some researchers have used sensory evaluation and analysis of essential quality components to evaluate gardenia black and green teas from Yichang and Yibin cities, identifying key aromatic elements without differentiating gardenia teas by their origin or production year [18,19,20]. Chen et al. examined the volatile aromatic compounds in gardenia-scented white tea made from a white tea base of varying storage durations [21], but their study was limited to a single control sample, lacking a thorough exploration of the aromatic changes during the scenting process. Additionally, the notable variations in quality and style among white teas from different years lead to diverse characteristics in the resulting gardenia white tea, complicating comparisons [22,23,24]. Thus, a more systematic approach is needed to analyze the aromatic profiles of flower teas combined with white tea of different storage lengths before and after the scenting process. This would enhance the categorization, differentiation, and assessment of the quality traits of gardenia flower tea based on storage time, providing valuable insights for practical production.

The fragrance of tea, a crucial quality factor, arises from a complex blend of numerous volatile organic compounds (VOCs) that work together to create the aroma [25]. While traditional sensory panels are essential for comprehensive assessments, they are inherently subjective and lack a solid quantitative basis [26]. As a result, the use of instrumental analysis has become vital. The technique of headspace solid-phase microextraction combined with gas chromatography–mass spectrometry (HS-SPME-GC-MS) is now considered the gold standard for profiling tea aromas. This method is solvent-free, requires only a small amount of sample, and effectively captures a wide range of trace-level VOCs, providing a highly accurate representation of the volatile profile [27]. Its adaptability has been proven across various tea types, including green (Longjing) [28], black (large-leaf black tea) [29], white (GABA white tea) [1], oolong (Tieguanyin, Rougui, Shuixian) [30], and specialty teas like Zijuan white tea [31] and Dian green tea [32], consistently revealing unique volatile signatures. The extensive data produced is well-suited for chemometric analysis. Multivariate techniques, particularly principal component analysis (PCA), simplify the intricate VOC data into a few key variables, allowing for objective differentiation of teas based on processing methods, geographic origins, or quality grades, thus providing a robust statistical framework for quality assurance [5,33].

White tea is primarily divided into two categories—fresh white tea and aged white tea. Fresh white tea is made from leaves that have undergone processing and have been stored for less than three years, while aged white tea consists of leaves that have been preserved for more than three years [22]. These categories display unique biochemical properties and flavor characteristics. Studies indicate that important biochemical elements, such as tea polyphenols, caffeine, amino acids, soluble sugars, and flavonoids, change considerably as white tea ages [34]. Sensory evaluations reveal that the scent of aged white tea transforms from a light, delicate aroma to a richer, more intricate fragrance. Furthermore, the color of the tea liquor shifts from pale yellow to a darker yellow, and the taste becomes more refined and full-bodied [35]. These variations in chemical makeup and sensory qualities between fresh and aged white tea lead to differences in aroma complexity, flavor depth, and aftertaste when used as a base for gardenia tea. This results in a range of styles that are difficult to compare directly. Consequently, this study employs HS-SPME-GC-MS technology along with multivariate statistical methods, including both statistical and univariate analyses, to investigate the differences in key volatile aroma compounds between the white tea bases and the scented gardenia white teas. The focus is on comparing the changes in aroma compounds between GNWT and NWT, as well as GAWT and AWT. The results of this research will enhance the understanding of the chemical foundations of the distinctive aroma of gardenia white tea, offering a theoretical basis for assessing quality and developing products related to gardenia flower white tea with varying storage times.

## 2. Materials and Methods

### 2.1. Materials and Reagents

This research involved the selection of two varieties of white tea as the foundation for the gardenia-scented white tea. One variety was made in the spring of 2025 (referred to as NWT), while the other was produced in the spring of 2015 (identified as AWT). Both types were produced by Cangnan (Han Tea) Agricultural Development Co., Ltd. (Wenzhou, China). The scenting process utilized gardenia blossoms that were picked at their peak bloom in early June 2025, employing the flower tea processing method to create the gardenia-scented white tea. The procedure for scenting the tea with gardenia follows the traditional flower tea scenting technique, as illustrated in Figure S1.

In June 2025, the process of infusing gardenia fragrance into white tea was carried out. Two varieties of gardenia white tea were created, referred to as GNWT for the freshly produced version and GAWT for the aged variant, with NWT and AWT serving as controls. To achieve the scenting, 30% of the raw white tea comprised gardenia flowers. The infusion took place over a static period of roughly 15 h, after which the tea was air-dried in an oven at 105 °C until the moisture level dropped to around 8%. This was followed by a second drying phase at 80 °C, ensuring the moisture content of the gardenia white tea fell below 6%.

Analytical-grade sodium chloride was obtained from Sinopharm Chemical Reagent Co., Ltd. (Shanghai, China), while chromatographic-grade n-hexane was sourced from Merck Darmstadt, Germany. in Germany. Standards were created with n-hexane and kept at a temperature of −20 °C, using chromatographic-grade material from BioBioPha/Sigma-Aldrich, (Saint Louis, MO, USA).

### 2.2. Instrument and Equipment

n-hexane, serving as the internal standard, was sourced from Sigma-Aldrich in Shanghai, China. The research utilized an Agilent 8890-7000D GC-MS/MS system, equipped with an Agilent DB-5MS column (Agilent, Santa Clara, CA, USA) measuring 30 m × 0.25 mm × 0.25 µm. Furthermore, a Retsch MM400 ball mill (Haan, Germany), a METTLER TOLEDO MS105DU analytical balance (Shanghai, China), and an Agilent extraction head featuring 120 µm DVB/CWR/PDMS (Santa Clara, CA, USA) were employed. For the headspace solid-phase microextraction (HS-SPME) process, a CTC Analytics SPME Arrow was implemented, alongside a CTC Analytics Fiber Conditioning Station for aging and a CTC Analytics agitator that served as the heating box for samples.

### 2.3. Sample Preparation and Extraction via Headspace Solid-Phase Microextraction (HS-SPME)

The materials were collected, weighed, quickly frozen in liquid nitrogen, and kept at −80 °C until needed. Subsequently, the samples were pulverized into a fine powder with the aid of liquid nitrogen.

For the extraction process, 500 mg of tea powder was transferred into a 20 mL headspace vial. A saturated solution of sodium chloride (NaCl) was then introduced, along with 20 μL of an internal standard solution (n-hexane at 10 μg/mL). Following this, automated headspace solid-phase microextraction (HS-SPME) was performed before proceeding with gas chromatography–mass spectrometry (GC-MS) analysis.

For HS-SPME procedures, the samples were stirred for 5 min at a stable temperature of 60 °C. Subsequently, a 120 µm DVB/CAR/PDMS extraction device was placed into the headspace of the sample vial and left to extract for 15 min. After extraction, the device was desorbed at 250 °C for 5 min prior to GC-MS analysis for separation and identification purposes. Before sampling, the extraction device underwent conditioning for 5 min at 250 °C in a Fiber Conditioning Station. It is noteworthy that new extraction devices are conditioned for 2 h in the Fiber Conditioning Station before they are used for extraction. The SPME Arrow utilized in this research offers a sensitivity that is tenfold greater than that of traditional SPME fibers.

### 2.4. GC-MS Conditions

After the sampling procedure, volatile organic compounds (VOCs) were desorbed from the SPME Arrow coating within the gas chromatography (GC) system’s injection port (Model 8890; Agilent) at 250 °C for 5 min. The analysis and measurement of VOCs utilized an Agilent Model 8890 GC paired with a 7000D mass spectrometer (Agilent), equipped with a 30 m × 0.25 mm × 0.25 μm DB-5MS capillary column (5% phenyl-polymethylsiloxane). Helium was used as the carrier gas, maintaining a flow rate of 1.2 mL/min. The injector was set to 250 °C. The oven temperature was programmed to start at 40 °C for 3.5 min, then increased by 10 °C/min to 100 °C, followed by a rise of 7 °C/min to 180 °C, and finally ramped up at 25 °C/min to reach 280 °C, where it remained for 5 min. Mass spectra were obtained using electron impact (EI) ionization at 70 eV. The quadrupole mass detector, ion source, and transfer line were maintained at temperatures of 150 °C, 230 °C, and 280 °C, respectively. The mass spectrometer operated in selected ion monitoring (SIM) mode for the detection and quantification of the target compounds.

### 2.5. Qualitative and Quantitative Analysis of Volatile

The aroma components in the tea samples were analyzed using a total ion chromatogram obtained through optimized GC-MS settings. A qualitative analysis of the volatile compounds was conducted with the aid of the MWGC database, which is based on the NIST library and enhanced with standard reference materials. Subsequently, the MassHunter (version 12.0) software was employed to process the mass spectral data, focusing on specific ions for the integration and calibration of the chromatographic peaks. The quantification of the compounds was performed by determining the ratio of the peak area for each identified volatile compound to that of the internal standard (n-hexane) for relative measurement. The equation for estimation is provided below.

$$X_{i}=\frac{V_{s}\;\times {\;C}_{s}}{M}\;\times \;\frac{I_{i}}{I_{s}}\;\times {\;10}^{-3}$$

X_i_ represents the amount of compound i present in the sample (measured in μg/g); V_s_ refers to the volume of the internal standard that has been introduced (in μL); C_s_ indicates the concentration of the internal standard (in μg/mL); M denotes the weight of the sample (500 mg); I_s_ is the peak area linked to the internal standard; and I_i_ is the peak area related to compound i within the sample.

### 2.6. Odor Activity Values (OAVs) Calculation

The relative odor activity value (rOAV) serves as a technique for evaluating the importance of flavor compounds in food by considering their sensory thresholds. This method aids in understanding how each aromatic component contributes to the overall scent profile of a sample. Recently, there has been a growing trend among researchers to use rOAV to identify key flavor compounds in different food items. Generally, an rOAV of 1 or higher suggests that the compound significantly influences the flavor of the sample. The formula for calculating rOAV can be found in relevant studies [36,37].

$$\mathrm{rOA}V_{i}=\frac{C_{i}}{T_{i}}$$

In this formula, rOAVi denotes the relative odor activity value for compound i, while Ci signifies the compound’s relative concentration (expressed in μg/g or μg/mL), and Ti indicates the threshold concentration of that compound (also in μg/g or μg/mL).

### 2.7. Statistical Analysis

Statistical comparisons involving NWT and GNWT, along with AWT and GAWT, were conducted utilizing SPSS 22 software (IBM, Armonk, NY, USA). Levene’s test was applied to check for variance homogeneity, while a t-test was used to determine significance, with a threshold of *p*-value < 0.05 for statistical significance. Additionally, orthogonal partial least discriminant analysis (OPLS-DA) was executed using SIMCA 14.1 software (Umetrics, Sweden). Furthermore, heatmaps and bar graphs were created using Origin2025b software (Originlab, Northampton, MA, USA).

## 3. Results

### 3.1. Identification of Volatile Compounds in Raw White Teas and Gardenia White Teas

**Figure 1:**
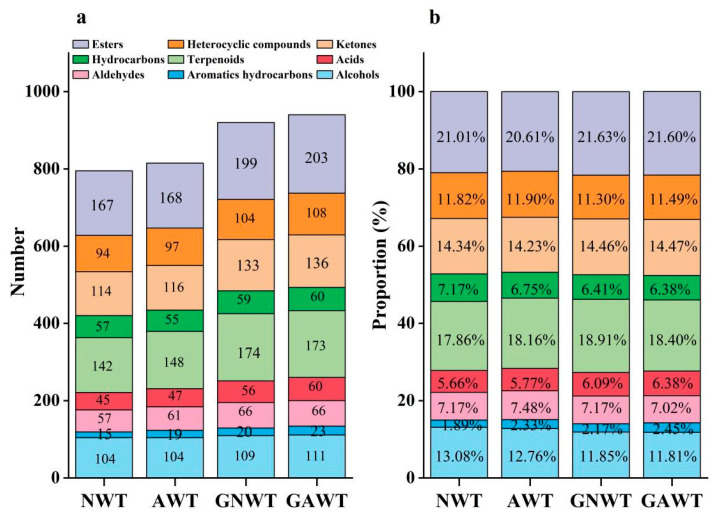
Information of volatile compounds in white tea base and gardenia white tea. (**a**) Specific number of volatile categories in raw white tea and gardenia white tea. (**b**) Proportion of volatile categories in white tea bases and gardenia white tea.

The identification of volatile aroma compounds in white tea bases and gardenia white teas was conducted using GC-MS analysis. In the non-scented white tea NWT, researchers found a total of 795 volatile compounds, which included 167 esters, 142 terpenoids, 114 ketones, 104 alcohols, 94 heterocyclic compounds, 57 aldehydes, 57 hydrocarbons, 45 acids, and 15 aromatic hydrocarbons. Conversely, the non-scented white tea AWT revealed 815 volatile compounds, comprising 168 esters, 148 terpenoids, 116 ketones, 104 alcohols, 97 heterocyclic compounds, 61 aldehydes, 59 hydrocarbons, 47 acids, and 19 aromatic hydrocarbons. The gardenia white tea GNWT was found to contain 920 volatile compounds, which included 199 esters, 174 terpenoids, 133 ketones, 109 alcohols, 104 heterocyclic compounds, 66 aldehydes, 59 hydrocarbons, 56 acids, and 20 aromatic hydrocarbons. Finally, the gardenia white tea GAWT showed 940 volatile compounds, consisting of 203 esters, 173 terpenoids, 136 ketones, 111 alcohols, 108 heterocyclic compounds, 66 aldehydes, 60 hydrocarbons, 60 acids, and 23 aromatic hydrocarbons (Figure 1a). The main categories across all samples were esters, terpenoids, ketones, and alcohols, which represented 21.01%, 17.86%, 14.34%, and 13.08% in NWT, 20.61%, 18.16%, 14.23%, and 12.76% in AWT, 21.63%, 18.91%, 14.46%, and 11.85% in GNWT, and 21.60%, 18.40%, 14.47%, and 11.81% in GAWT, respectively (Figure 1b), with all categories surpassing 10%. This aligns with earlier studies [38,39], which indicated that the volatile components of fresh gardenia flowers are mainly ester compounds, known for their sweet fruity and floral scents, forming the foundation of the fragrance of gardenia blooms.

### 3.2. Changes in Volatile Compounds After Scenting with Gardenia Flowers

**Figure 2:**
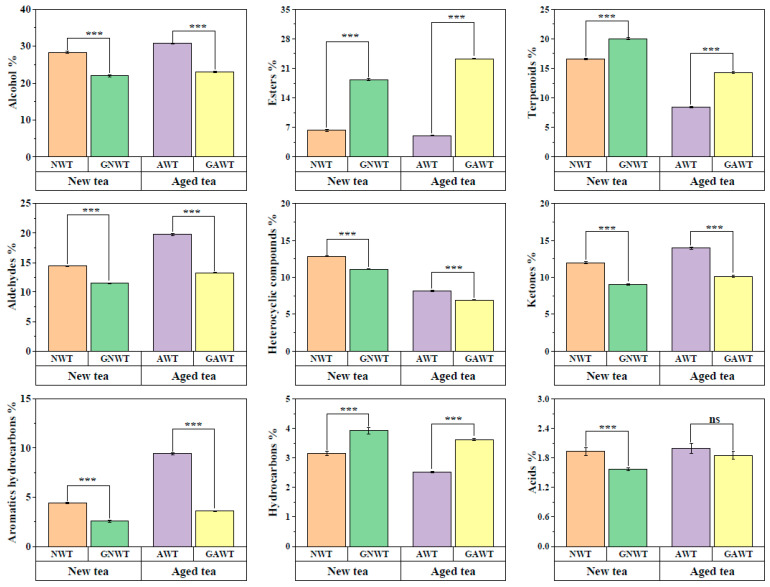
Paired comparison of the proportional content of each compound category between NWT vs. GNWT, and AWT vs. GAWT. ‘***’ indicate significant difference at *p* < 0.001 and ‘ns’ means no significant difference between treatments.

To gain insights into the alterations in aromatic compounds in gardenia white tea following the scenting process, a paired comparison method was utilized to statistically evaluate the differences in the proportions of various aromatic constituents before and after the scenting process, using two sets of raw white teas and gardenia white teas. The findings revealed that the patterns of change in aromatic components in gardenia white tea derived from different white tea bases (NWT and AWT) were similar. Both types exhibited a notable rise in the levels of esters, terpenoids, and hydrocarbons, while there was a marked decline in the levels of alcohols, aldehydes, heterocyclic compounds, ketones, aromatic hydrocarbons, and acids (Figure 2). These results support previous studies that highlight the importance of reducing alcohol compounds and increasing ester compounds in the aroma transformation during scented tea production [40,41]. Significantly, the ester content saw the largest increase, climbing from 6.29% and 5.01% in NWT and AWT, respectively, to 18.33% and 23.27% in GNWT and GAWT. The terpenoid levels rose from 16.57% in NWT and 8.41% in AWT to 20.01% in GNWT and 14.32% in GAWT. In contrast, alcohols experienced the most considerable reduction, with their proportions falling from 28.39% and 30.77% in NWT and AWT to 21.95% and 22.99% in GNWT and GAWT, respectively. The aldehyde levels decreased from 14.39% and 19.74% in NWT and AWT to 11.53% and 13.26% in GNWT and GAWT, respectively. Furthermore, ketone levels dropped from 11.98% in NWT to 9.03% in GNWT, and from 13.95% in AWT to 10.15% in GAWT.

### 3.3. Analysis of Key Aromatic Compounds in GNWT Prepared by NWT

#### 3.3.1. OPLS-DA Modeling in the Volatile Compounds

**Figure 3:**
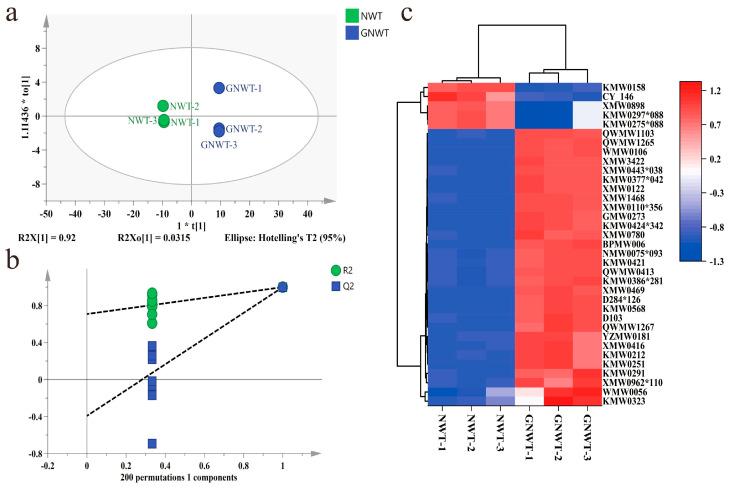
Multivariate analysis results of volatile compounds of NWT and GNWT. (**a**) Score plot of OPLS−DA (R^2^Y = 1, Q^2^ = 0.999). (**b**) Plot of 200 permutation tests (R^2^Y = 0.709, Q^2^ = −0.392). (**c**) Heatmap constructed with 35 key differential volatiles with VIP > 2.0 and *p* < 0.05 in NWT and GNWT. Note: in Figure 3c, compounds’ codes correspond to the following compound names. KMW0275*088 = (*2R*,*5S*)-5-Ethenyl-2-methyl-5-(prop-1-en-2-yl)cyclohexane-1,2-diol, KMW0323 = phenylmethanol, KMW0297*088 = 2-[(*2S*,*5S*)-5-ethenyl-5-methyloxolan-2-yl]propan-2-ol, WMW0056 = (*E*)-non-4-enal, XMW0898 = 4-(2-methylprop-yl)pyrimidine, CY-146 = 4,4,6-trimethylcyclohex-2-en-1-one, XMW0962*110 = benzyl-2-methylbutanoate, KMW0251 = 2,6-dimethylhept-5-enal, KMW0291 = linalool, KMW0212 = 2-phenylacetaldehyde, XMW0416 = 5-butylcyclohexa-1,3-diene, YZMW0181 = (*Z*)-2-(1-pentenyl)-Furan, QWMW1267 = 3-methylbutyl2-phenylacetate, XMW0780 = (*3E*,*5E*)-2,6-dimethylocta-1,3,5,7-tetraene, D103 =(2-methyl-1-phenylpropan-2-yl)butanoate, KMW0568 = 6-pentyloxan-2-one, D284*126 = 2-phenylethyl2-methylbutanoate, KMW0158 = Benzaldehyde, XMW0469 = (*1R*,*3aS*,*8aS*)-1,4-dimethyl-7-propan-2-yl-1,2,3,3a,6,8a-hexahydroazulene, KMW0386*281 = *cis*-(*2R*,*5S*)-2-methyl-5-prop-1-en-2-ylcyclohexan-1-one, NMW0075*093 = 1-methyl-4-propan-2-ylidenecyclohexan-1-ol, KMW0421 = methyl2-hydroxybenzoate, QWMW0413 = 2-butylpyridine, KMW0424*342 = hexyl2-methylbutanoate, GMW0273 = 2-hexylcyclopentan-1-one, BPMW006 = diethyl2-methyl-3-oxobutanedioate, XMW0122 = (2-methyl-3-prop-1-en-2-ylcyclohexyl)acetate, QWMW1103 = hexyl(*E*)-2-methylbut-2-enoate, KMW0377*042 = [(*Z*)-hex-3-enyl]2-methylbutanoate, XMW3422 = 2-pentylpyrazine, XMW0110*356 = 4,6-dimethyldodecane, XMW1468 = (2,2,6-trimethyl-1-bicyclo[4.1.0]heptanyl)methanol, QWMW1265 = 1-isothiocyanato-3-methylsulfanylpropane, XMW0443*038 = (*E*)-2,6-Dimethylocta-3,7-diene-2,6-diol, WMW0106 = [(*Z*)-non-6-enyl] acetate.

OPLS-DA is widely applied in tea analysis for classifying and identifying aromas, components, and quality, as well as for selecting features and identifying sources of variation [42]. In the context of OPLS-DA modeling, R^2^X and R^2^Y values nearing 1 indicate an excellent fit for the model. The model’s reliability is further confirmed through a permutation test, which is validated if all permuted Q^2^ values are less than the original Q^2^, or if the intercept of the Q^2^ regression line on the Y-axis is less than or equal to 0 [43]. To distinguish between NWT and GNWT, an OPLS-DA model was created using a data matrix that included 980 volatile compounds from six samples. As shown in Figure 3a, a clear distinction between NWT and GNWT was observed. The model parameters (R^2^Y = 1, Q^2^ = 0.999) demonstrated strong explanatory and predictive power. To evaluate the model’s robustness, 200 permutation tests were performed in Figure 3b, yielding R^2^ and Q^2^ values of (0, 0.709) and (0, −0.392), respectively, which confirmed the model’s robustness and ruled out overfitting.

#### 3.3.2. Analysis of Differential Volatile Compounds in NWT and GNWT

To delve deeper into the significant volatile compounds that differentiate NWT from GNWT, an analysis of variable importance in projection (VIP) was performed. Typically, a VIP value exceeding 1 indicates that the variable is essential for classification purposes [44]. In this investigation, 35 volatile compounds were identified with VIP values greater than 2.0 and *p*-values below 0.05. To illustrate the distinctions between NWT and GNWT, a heatmap analysis was executed, as shown in Figure 3c. The red squares signify elevated concentrations, while the blue squares denote reduced levels. Five volatile compounds, including 2-[(*2S*,*5S*)-5-ethenyl-5-methyloxolan-2-yl]propan-2-ol, benzaldehyde, 4,4,6-trimethylcyclohex-2-en-1-one, 2-(5-ethenyl-5-methyloxolan-2-yl)propan-2-ol, 4-(2-methylpropyl)pyrimidine were found to be more abundant in NWT compared to GNWT. Conversely, 30 volatile compounds, such as linalool, phenylmethanol, (*E*)-non-4-enal, 2-phenylacetaldehyde, methyl 2-hydroxybenzoate, hexyl (*E*)-2-methylbut-2-enoate, 2-pentylpyrazine and [(*Z*)-non-6-enyl] acetate, exhibited higher concentrations in GNWT than in NWT.

#### 3.3.3. OAV Analysis of Volatile Compounds in NWT and GNWT

OAV serves as a crucial metric for assessing the role of volatile compounds in the overall aroma. Typically, volatile compounds with an OVA of 1 or higher are regarded as significant contributors to the complete aroma profile [45]. In this research, the OVA values for 16 distinct aroma components in NWT and GNWT were determined based on established thresholds and descriptions found in the existing literature [46,47]. From those with OVA values exceeding one (Table 1), 14 aroma compounds were identified for further analysis. Notably, 1-isothiocyanato-3-(methylthio)-2-propane (OAV = 0.03), 6-pentyloxan-2-one (OAV = 0.41), and [(*Z*)-non-6-enyl] acetate (OAV = 0.08) were the only three compounds with OAVs below one in NWT, while the remaining 11 volatile compounds had OAVs above one in both NWT and GNWT. Additionally, benzaldehyde(OAV = 25.83), 2-(5-ethenyl-5-methyloxolan-2-yl)propan-2-ol (OAV = 28.80), and 2-[(*2S*,*5S*)-5-ethenyl-5-methyloxolan-2-yl]propan-2-ol (OAV = 48.51) exhibited higher OAVs in NWT compared to GNWT, whereas the other 11 volatile compounds had significantly elevated OAVs in GNWT relative to NWT. Notably, [(*Z*)-non-6-enyl] acetate (OAV-GNWT = 13973.95) and 2-pentylpyrazine (OAV-GNWT = 1040.56) recorded OAVs exceeding 1000 in GNWT, while linalool (OAV-GNWT = 4858.46, OAV-NWT = 2096.61) and (*E*)-non-4-enal (OAV-GNWT = 8625.95, OAV-NWT = 7951.62) also surpassed 1000 in both regions. These compounds are deemed essential for differentiating the aroma profiles of GNWT and NWT.

#### 3.3.4. Analysis of Key Aromatic Compounds in GNWT

**Figure 4:**
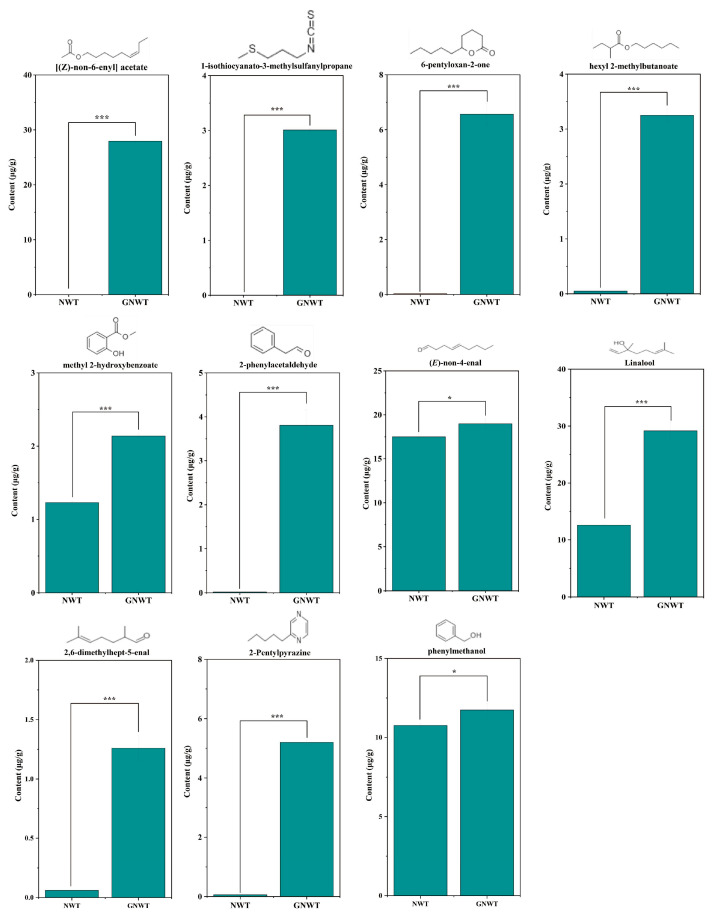
Structure and contents of 11 key volatile compounds significantly higher in GNWT than in NWT. ‘*’, and ‘***’ indicate significant difference at *p* < 0.05, and *p* < 0.001, respectively.

To delve deeper into the distinctive aromatic elements of GNWT, a comparative study was performed on 14 essential aroma compounds, focusing on those that exhibited significant increases post-scenting. This analysis identified 11 key aroma compounds, [(*Z*)-non-6-enyl] acetate, linalool, (*E*)-non-4-enal, 2-phenylacetaldehyde, methyl2-hydroxybenzoate, 2-pentylpyrazine, 2,6-dimethylhept-5-enal, butanoic acid 2-methyl-hexylester, 1-isothiocyanato-3-(methylthio)-propane, tetrahydro-6-pentyl-2H-Pyran-2-one, and phenylmethanol. The chemical structures and statistical data regarding the concentrations of these compounds are illustrated in Figure 4. It is evident that (*E*)-non-4-enal, linalool, and phenylmethanol are the most concentrated aroma compounds in NWT, with values of 17.49, 12.58, and 10.77 µg/g, respectively. In contrast, the remaining eight compounds have notably lower concentrations, primarily under 0.05 µg/g, with the highest reaching only 1.23 µg/g. Conversely, GNWT displays a richer array of aroma compounds, with 11 substances ranging from 1.26 to 29.15 ug/g. The three most concentrated compounds in GNWT are linalool (29.15 µg/g), [(*Z*)-non-6-enyl] acetate (27.95 µg/g), and (*E*)-non-4-enal (18.98 µg/g), all significantly surpassing their counterparts in NWT. Remarkably, the concentration of [(*Z*)-non-6-enyl] acetate surged from 4.7 × 10^−4^ µg/g in NWT to 27.95 µg/g in GNWT, marking an increase of over 50,000 times. Linalool stands out as the dominant aromatic compound in floral teas, known for its strong and recognizable scent [37]. Furthermore, [(*Z*)-non-6-enyl] acetate, along with its alcohol precursor, contributes a delicate fruity aroma and honeydew flavor, enhancing the tea’s overall sweetness [48]. (*E*)-non-4-enal is acknowledged for its role in imparting fruity fragrances, offering a distinct green fruit scent to the tea leaves [49]. These compounds are generated during the scented tea processing through enzymatic or oxidative reactions, working together to produce a unique aroma characterized mainly by floral and fruity notes. This intricate interplay allows for the differentiation of aroma profiles between GNWT and NWT.

### 3.4. Analysis of Key Aromatic Compounds in GAWT Prepared by AWT

#### 3.4.1. OPLS-DA Analysis in the Volatile Compounds

**Figure 5:**
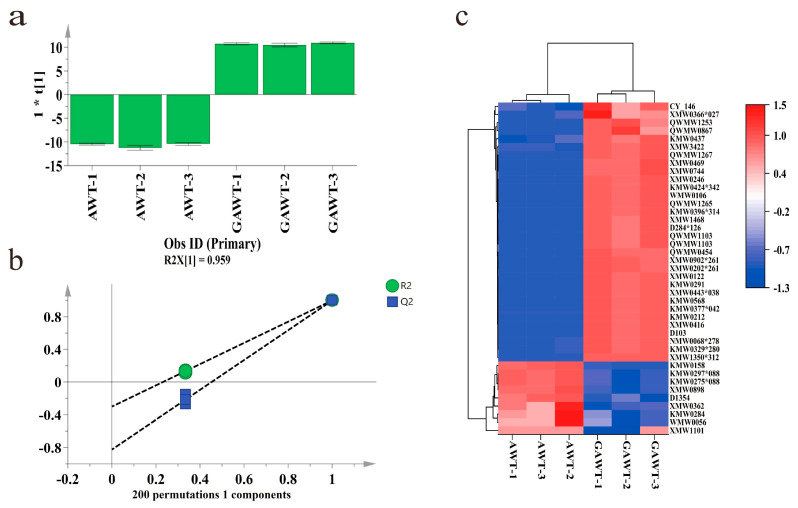
Multivariate analysis results of volatile compounds of AWT and GAWT. (**a**) Score plot of OPLS-DA (R^2^Y = 0.999, Q^2^ = 0.999). (**b**) Plot of 200 permutation tests (R^2^Y = −0.301, Q^2^ = −0.825). (**c**) Heatmap constructed with 40 key differential volatiles with VIP > 2.0 and *p* < 0.05 in AWT and GAWT. Note: in Figure 5c, compounds’ codes correspond to the following compound names. XMW1101 = 2,5-diisopropyl-1,4-dimethylbenzene, WMW0056 = (*E*)-4-Nonenal, XMW1350*312 = 1,2,7-trimethylnaphthalene, KMW0284 = 2-phenylethanol, XMW0366*027 = 1,2,3,5-tetramethylbenzene, XMW0362 = 4,6,6-trimethyloxan-2-one, CY-146 = 4,4,6-trimethylcyclohex-2-en-1-one, D1354 = octan-4-ol, QWMW0867 = ethyl2-hydroxy-3-methylbutanoate, KMW0437 = 2-Phenylethylacetate, XMW0898 = 4-(2-methylpropyl)pyrimidine, KMW0297*088 = 2-[(*2R*,*5R*)-5-ethenyl-5-methyloxolan-2-yl]propan-2-ol, KMW0275*088 = 2-[(*2R*,*5S*)-5-ethenyl-5-methyloxolan-2-yl]propan-2-ol, QWMW1253 = 2-methylpropylbut-2-enoate, QWMW1103 = hexyl(*E*)-2-methylbut-2-enoate, XMW0744 = (*2E*,*6Z*)-dodeca-2,6-dienal, XMW0469 = (*1R*,*3aS*,*8aS*)-1,4-dimethyl-7-propan-2-yl-1,2,3,3a,6,8a-hexahydroazulene, XMW3422 = 2-Pentylpyrazine, D284*126 = 2-phenylethyl 2-methylbutanoate, QWMW1267 = 3-methylbutyl 2-phenylacetate, XMW1468 = (2,2,6-trimethyl-1-bicyclo [4.1.0]heptanyl)methanol, KMW0158 = benzaldehyde, KMW0329*280 = camphor, KMW0396*314 = ethyl(*E*)-oct-2-enoate, QWMW1265 = 1-isothiocyanato-3-methylsulfanylpropane, WMW0106 = [(*Z*)-non-6-enyl]acetate, D103 = (2-methyl-1-phenylpropan-2-yl)butanoate, KMW0424*342 = hexyl2-methylbutanoate, NMW0068*278 = (4-methylphenyl)acetate, XMW0416 = 5-butylcyclohexa-1,3-diene, XMW0246 = 3-methyl-3-(4-methylpent-3-en-1-yl)oxirane-2-carbaldehyde, XMW0202*261 = (*6Z*)-2,6-dimethylocta-2,6-diene, XMW0902*261 = (*2E*,*6E*)-2,6-dimethyocta-2,6-diene, KMW0212 = 2-phenylacetaldehyde, XMW0443*038 = (*3E*)-2,6-dimethylocta-3,7-diene-2,6-diol, KMW0377*042 = [(*Z*)-hex-3-enyl]2-methylbutanoate, QWMW0454 = 2-(2-methylpropyl)pyridine, KMW0291 = linalool, XMW0122 = (1r,2r,3r)-3-isopropenyl-2-methylcyclohexan-1-ol, KMW0568 = 6-pentyloxan-2-one.

To differentiate between AWT and GAWT, an OPLS-DA model was created. As illustrated in Figure 5a, there was a distinct separation observed between AWT and GAWT. The parameters of the model (R^2^Y = 0.999, Q^2^ = 0.999) indicated a high level of explanatory and predictive capability. To assess the model’s reliability, 200 permutation tests were conducted. The findings revealed R2 and Q2 values of (0, −0.301) and (0, −0.825), respectively, which confirmed the model’s reliability and ruled out overfitting (Figure 5b).

#### 3.4.2. Analysis of Differential Volatile Compounds in AWT and GAWT

Furthermore, a VIP analysis was conducted to delve deeper into the significant volatile compounds that set AWT apart from GAWT. A total of 40 volatile compounds were identified, each with VIP values exceeding 2 and *p*-values below 0.05. To highlight the differences between AWT and GAWT, a heatmap analysis was carried out, as shown in Figure 5c. Among these compounds, nine compounds, such as 1,4-dimethyl-2,5-bis(1-methylethyl)-benzene, (*E*-non-4-enal, and 2-phenylethanol, were found in higher amounts in AWT relative to GAWT. In contrast, 31 volatile compounds, including key substances like linalool, Camphor, and 2-phenylethyl acetate, were present in greater quantities in GAWT compared to AWT.

#### 3.4.3. OAV Analysis of Volatile Compounds in AWT and GAWT

Following the established criteria and descriptions of aromatic elements found in previous studies [46,47], the OVA values for 16 unique aroma substances in AWT and GAWT were determined. The analysis revealed that 16 aroma substances had OVA values greater than one (Table 2). Among these, only 6-pentyloxan-2-one (OAV = 0.57) and [(*Z*)-non-6-enyl] acetate (OAV = 0.07) showed OAVs below one in AWT, while the other 14 volatile substances had OAVs exceeding one in both AWT and GAWT. Notably, compounds such as octan-4-ol (OAV = 19.56), benzaldehyde (OAV=48.91), 2-(5-ethenyl-5-methyloxolan-2-yl)propan-2-ol(OAV = 15.26), 2-[(*2S*,*5S*)-5-ethenyl-5-methyloxolan-2-yl]propan-2-ol (OAV = 25.71), 2-phenylethanol (OAV = 472.60), and (*E*)-non-4-enal (OAV = 10408.68) displayed higher OAVs in AWT than in GAWT, while the other 10 volatile substances had significantly elevated OAVs in GAWT compared to AWT. It is noteworthy that [(*Z*)-non-6-enyl] acetate (OAV-GAWT = 15022.70) and linalool (OAV-GAWT = 4425.37) had OAVs exceeding 1000 in GAWT when compared to AWT. Furthermore, (*E*)-non-4-enal (OAV-GNWT = 9204.56, OAV-NWT = 10408.68) also recorded OAVs above 1000 in both GAWT and AWT. These substances are considered essential for distinguishing the aroma characteristics between GAWT and AWT.

#### 3.4.4. Analysis of Key Aromatic Compounds in GAWT

**Figure 6:**
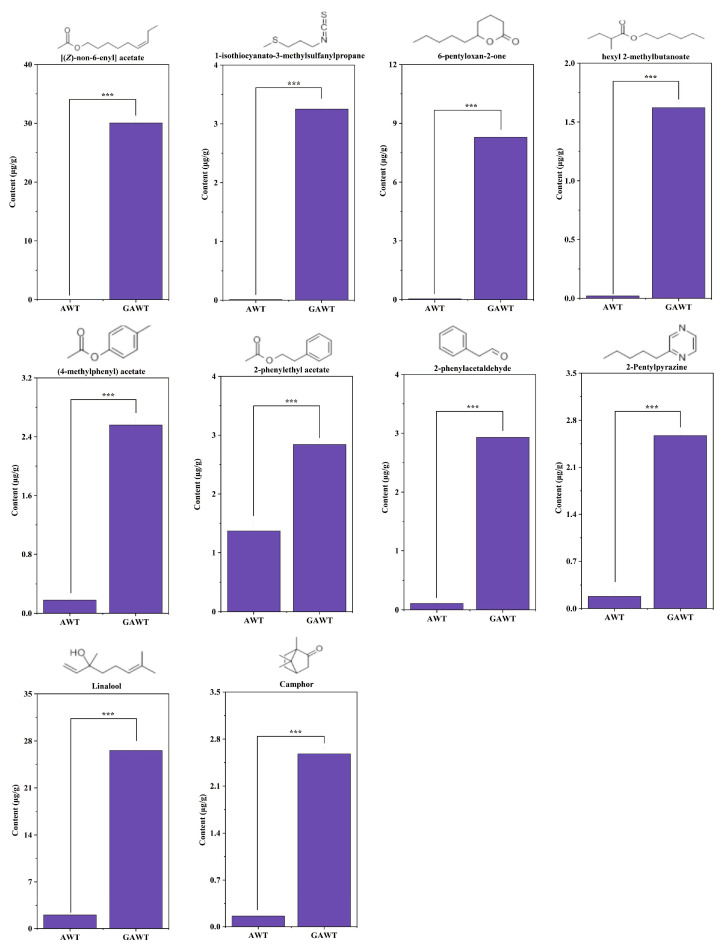
Structure and contents of 10 key volatile compounds significantly higher in GAWT than in AWT. ‘***’ indicate significant difference at *p* < 0.001.

To investigate the notable rise in volatile compound levels in GAWT after the scenting process, a comparative study was conducted on 16 essential aroma compounds to identify the distinctive aromatic elements of GAWT. This analysis revealed 10 specific aroma compounds, which are [(*Z*)-non-6-enyl] acetate, Linalool, 2-phenylacetaldehyde, 2-pentylpyrazine, Butanoic acid, Camphor, 1-isothiocyanato-3-(methylthio)-Propane, tetrahydro-6-pentyl-2H-Pyran-2-one, 2-methyl-hexyl ester, (4-methylphenyl) acetate, and 2-phenylethyl acetate. The structural formulas and statistical data for these compounds are illustrated in Figure 6. It is evident that all 10 compounds are present in low amounts in AWT, with concentrations ranging from 1.30 × 10^−4^ µg/g to 2.05 µg/g. In contrast, the levels of these aroma compounds in GAWT are significantly higher, with concentrations for the 10 compounds falling between 1.62 and 30.05 µg/g. Each of these compounds showed a marked increase in GAWT compared to AWT. Notably, [(*Z*)-non-6-enyl] acetate (30.05 µg/g) and linalool (26.56 µg/g) are among those exceeding 10 µg/g. The concentration of (*Z*)-6-Nonen-1-ol acetate experienced a dramatic rise from 1.3 × 10^−4^ µg/g in AWT to 30.05 µg/g in GAWT, indicating an increase of over 230,000 times. These changes in compound concentrations, especially the significant variations in key compounds like [(*Z*)-non-6-enyl] acetate and linalool, are likely crucial in differentiating the aroma profiles of GAWT and AWT.

## 4. Discussion

Aroma is a crucial quality factor in the scenting process of scented tea, serving as an important indicator for measuring and evaluating tea quality [50]. This study compares the volatile components of white tea leaves before and after the scenting process with gardenia flowers across different years, revealing significant differences in the content of aromatic compounds. By utilizing OPLS-DA, VIP, and OAVs, we identified 11 and 10 key aroma compounds in GNWT and GAWT, respectively. The main common compounds include [(*Z*)-non-6-enyl] acetate, linalool, 2-phenylacetaldehyde, 2-pentylpyrazine,1-isothiocyanato-3-methylsulfanylpropane, 6-pentyloxan-2-one and 2-methyl-hexyl acetate. The content of these compounds is significantly higher than that in the corresponding year tea bases. Notably, the increase in the content of [(*Z*)-non-6-enyl] acetate was the most significant, with increases in GNWT and GAWT exceeding 50,000 times and 230,000 times, respectively. Previous research indicates that the green-herb and leafy notes from [(*Z*)-non-6-enyl] acetate impart freshness [51]. Linalool contributes dominant floral and sweet almond tones [52]. 2-phenylacetaldehyde possesses a complex aroma profile characterized by green, sweet, floral, and fruity notes [53]. 2-Pentylpyrazine is a low-threshold alkylpyrazine with a subtle green-pepper-earthy aroma [54]. 1-isothiocyanato-3-methylsulfanylpropane introduces a mild spicy and sulfurous edge [55]. 2-Methylhexyl acetate carries a fresh aroma reminiscent of sweet fruit notes, including green apple, pear, banana, and peach [56]. Collectively, these compounds shape the characteristic aroma of scented teas.

In this study, a comparison of the aroma components of gardenia-scented white tea to the tea base revealed a decreasing trend in the content of certain compounds. The GNWT exhibited a reduction in the levels of three aroma components relative to the tea base: benzaldehyde, 2-(5-ethenyl-5-methyloxolan-2-yl)propan-2-ol and 2-[(2S,5S)-5-ethenyl-5-methyloxolan-2-yl]propan-2-ol. Benzaldehyde is known to contribute to the sweet, fruity and floral aromas in tea, while 2-(5-ethenyl-5-methyloxolan-2-yl)propan-2-ol is associated with floral notes [57,58]. 2-[(*2S*,*5S*)-5-ethenyl-5-methyloxolan-2-yl]propan-2-ol, on the other hand, combines floral, sweet, and baked/roasted scents [59]. Therefore, the observed decrease in the content of these three components may have a detrimental effect on the overall aroma profile of Gardenia tea. In the GAWT, the content of six aroma compounds has decreased to varying degrees compared to the tea base prior to scenting. These compounds include benzaldehyde, 2-(5-ethenyl-5-methyloxolan-2-yl)propan-2-ol, 2-[(*2S*,*5S*)-5-ethenyl-5-methyloxolan-2-yl]propan-2-ol, 2-phenylethanol, (*E*)-non-4-enal, and octan-4-ol, with the first three compounds also present in GNWT. 2-phenylethanol is commonly described in tea as ‘floral-sweet-soft,’ making it one of the most characteristic floral base notes in scented tea [60]. (*E*)-non-4-enal contributes a secondary layer of green-fatty notes to the scented tea, enhancing the natural feel when utilized in moderation [61]. Additionally, octan-4-ol imparts a soft citrus-fatty aftertaste in the later stages, prolonging the aroma and balancing the overall taste [62]. Thus, even a modest reduction in the levels of these three compounds can significantly impact the aroma of scented tea. However, the aromatic characteristics of the scent result from the synergistic interaction of multiple components, necessitating comprehensive control [63]. In future, we will employ tea sensory evaluation to elucidate the contribution of each major aroma component to the fragrance of gardenia-scented white tea, aiming to provide theoretical support for the quality optimization and product development of gardenia white tea.

## 5. Conclusions

This research characterized the volatile compounds of gardenia white tea (GWT) through HS-SPME-GC-MS and multivariate analysis. The findings indicated that the main chemical classes in the samples of NWT, AWT, GNWT, and GAWT were esters, terpenoids, ketones, and alcohols. Further multivariate analysis pinpointed 11 significant volatile compounds (such as linalool, [(*Z*)-non-6-enyl] acetate, and (*E*)-non-4-enal) and 10 additional key compounds (including linalool, [(*Z*)-non-6-enyl] acetate, and 1-isothiocyanato-3-(methylthio)-2-Propane) with VIP values exceeding 2 and OAVs greater than 1, which helped differentiate the aroma profiles of GNWT from NWT and GAWT from AWT. The concentrations of these compounds were notably higher in GWT (both GNWT and GAWT) compared to WTB (NWT and AWT). Moreover, three volatile compounds in GNWT and six in GAWT exhibited a decline when compared to NWT and AWT, respectively. These compositional differences are believed to account for the distinct aroma variations between GWT and WTB. The outcomes of this research provide a theoretical basis and practical guidance for enhancing processes and elevating the quality of gardenia tea.

## Figures and Tables

**Table 1:** OAVs (above 1) and VIP values (above 2) of differential aroma components between NWT and GNWT.

Compounds	CAS	Threshold (μg/g)	ROAV Value		VIP Value	Odor
			NWT	GNWT		
1-isothiocyanato-3-(methylthio)- 2-Propane	505-79-3	0.005	0.03 ± 0.00 b	602.64 ± 5.78 a	3.84	earthy, vegetable, sulfury, horseradish
6-pentyloxan-2-one	705-86-2	0.066	0.41 ± 0.05 b	99.43 ± 5.79 a	5.65	creamy, coconut, fruity
hexyl 2-methyl butanoate	10032-15-2	0.022	2.35 ± 0.18 b	147.54 ± 5.61 a	3.96	green, waxy, fruity, apple, spicy, tropical
2,6-dimethylhept-5-enal	106-72-9	0.016	3.57 ± 0.21 b	78.93 ± 6.99 a	2.43	fresh, ozonous, melon, fresh air, sweet, green
2-phenylacetaldehyde	122-78-1	0.0063	3.94 ± 0.09 b	604.99 ± 54.89 a	4.30	floral, honey, rose, cherry
2-pentylpyrazine	6303-75-9	0.005	11.02 ± 0.24 b	1040.56 ± 22.00 a	5.02	roasted, nutty, popcorn-like
benzaldehyde	100-52-7	0.350	25.83 ± 0.32 a	15.97 ± 0.39 b	4.11	sweet, bitter, almond, cherry
2-(5-ethenyl-5-methyloxolan-2-yl)propan-2-ol	5989-33-3	0.320	28.80 ± 0.25 a	25.04 ± 1.58 b	2.31	earthy, floral, sweet, woody
methyl 2-hydroxybenzoate	119-36-8	0.040	30.84 ± 0.54 b	53.40 ± 0.76 a	2.10	caramel, pepperminty
2-[(*2S*,*5S*)-5-ethenyl-5-methyloxolan-2-yl]propan-2-ol	34995-77-2	0.190	48.51 ± 0.43 a	42.16 ± 2.67 b	2.31	flowery
[(*Z*)-non-6-enyl] acetate	76238-22-7	0.002	0.08 ± 0.00 b	13973.95 ± 146.92a	11.69	melon, honeydew, cantaloupe, green, tropical, pear, kiwi, metallic
phenylmethanol	100-51-6	0.100	107.74 ± 1.07 b	117.37 ± 4.25 a	2.04	floral, rose, phenol, balsamic
linalool	78-70-6	0.006	2096.61 ± 10.94 b	4858.46 ± 254.34 a	8.98	floral, green
(*E*)-non-4-enal	2277-16-9	0.002	7951.62 ± 141.75 b	8625.95 ± 243.96 a	2.56	fruity

Note: different lowercase letters (a and b) indicate significant difference at *p* < 0.05.

**Table 2:** OAVs (above 1) and VIP values (above 2) of differential aroma components between AWT and GAWT.

Compounds	CAS	Threshold (μg/g)	ROAV Value		VIP Value	Odor
			AWT	GAWT		
octan-4-ol	589-62-8	0.400	19.56 ± 0.60 a	9.50 ± 1.40 b	3.94	sweet, woody, waxy
2-(5-ethenyl-5-methyloxolan-2-yl)propan-2-ol	5989-33-3	0.320	15.26 ± 0.22 a	10.36 ± 0.42 b	2.46	earthy, floral, sweet, woody
2-phenylethyl acetate	103-45-7	0.249	5.47 ± 0.64 b	11.37 ± 0.29 a	2.38	rose, honey, tobacco
2-[(2S,5S)-5-ethenyl-5-methyloxolan-2-yl]propan-2-ol	34995-77-2	0.190	25.71 ± 0.37 a	17.45 ± 0.70 b	2.46	flowery
benzaldehyde	100-52-7	0.350	48.91 ± 1.08 a	20.36 ± 0.53 b	6.23	sweet, bitter, almond, cherry
hexyl 2-methylbutanoate	10032-15-2	0.022	1.01 ± 0.05 b	73.43 ± 2.40 a	2.49	green, waxy, fruity, apple, spicy, tropical
(4-methylphenyl) acetate	140-39-6	0.025	7.20 ± 0.73 b	102.41 ± 3.06 a	3.04	narcissus, phenol, animalic
6-pentyloxan-2-one	705-86-2	0.066	0.57 ± 0.03 b	125.61 ± 2.50 a	5.66	creamy, coconut, fruity
camphor	76-22-2	0.016	10.07 ± 0.88 b	161.43 ± 6.18 a	3.07	camphor
2-phenylethanol	60-12-8	0.140	472.60 ± 16.39 a	415.31 ± 11.33 b	5.41	fruity, rose, sweet, apple
2-phenylacetaldehyde	122-78-1	0.006	17.81 ± 0.86 b	464.95 ± 12.51 a	3.31	floral, honey, rose, cherry
2-Pentylpyrazine	6303-75-9	0.005	47.88 ± 0.93 b	513.89 ± 13.10 a	3.05	roasted, nutty, popcorn-like
1-isothiocyanato-3-(methylthio)- 2-Propane	505-79-3	0.005	3.45 ± 0.87 b	650.15 ± 23.32 a	3.55	earthy, vegetable, sulfury, horseradish
Linalool	78-70-6	0.006	342.31 ± 5.30 b	4425.37 ± 102.98 a	9.76	floral, green
(*E*)-non-4-enal	2277-16-9	0.002	10408.68 ± 390.17 a	9204.56 ± 255.65 b	3.09	fruity
[(*Z*)-non-6-enyl] acetate	76238-22-7	0.002	0.07 ± 0.00 b	15022.70 ± 527.06 a	10.81	melon, honeydew, cantaloupe, green, tropical, pear, kiwi, metallic

Note: different lowercase letters (a and b) indicate significant difference at *p* < 0.05.

## Data Availability

The original contributions presented in the study are included in the article; further inquiries can be directed to the corresponding author.

## Supplementary Materials

The following supporting information can be downloaded at https://www.mdpi.com/article/10.3390/metabo15120785/s1, Figure S1: The processing flowchart of gardenia-scented white tea by traditional scenting process.
