# Supplementary material for: Investigation of Volatile Compounds in Varied Types of Gardenia White Teas Utilizing HS–SPME–GC–MS and Multivariate Analysis
Source: Metabolites. 2025 Dec 5;15(12):785. doi: 10.3390/metabo15120785 (PMC12734676; doi:10.3390/metabo15120785)
Supplement: Supplementary file 1 [file metabolites-15-00785-s001.zip › metabolites-3995539-supplementary.pdf]

Supplementary material

1. Fig. S1 The processing flowchart of gardenia scented white tea by traditional scenting process

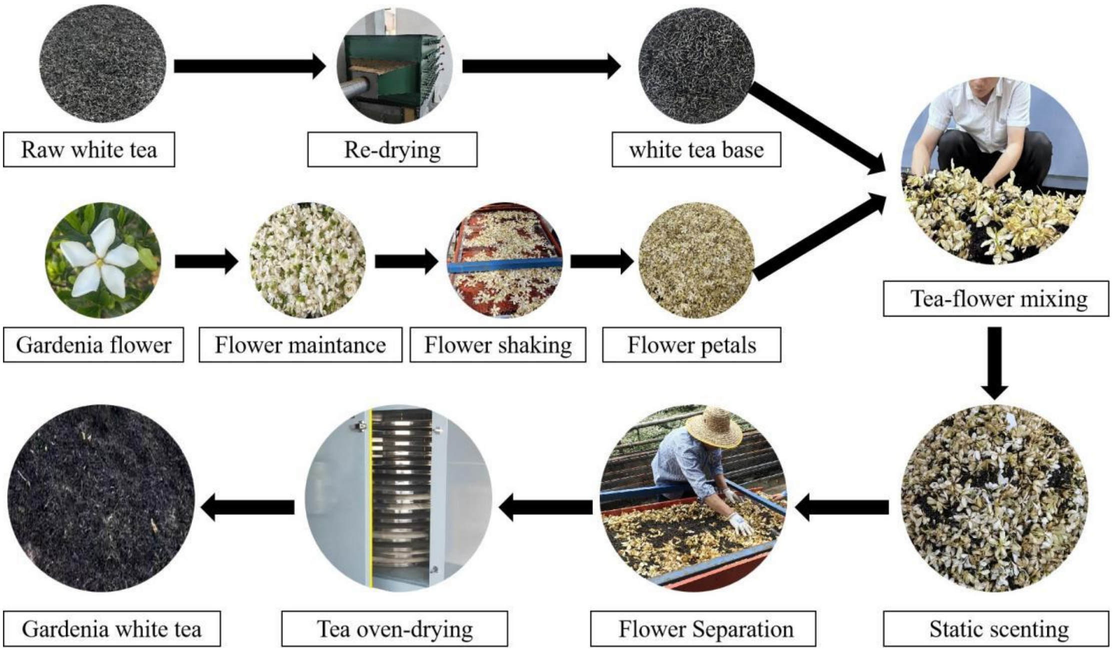

Fig. S1 The processing flowchart of gardenia scented white tea by traditional scenting process
